# Association of Arch Stiffness with Plantar Impulse Distribution during Walking, Running, and Gait Termination

**DOI:** 10.3390/ijerph17062090

**Published:** 2020-03-21

**Authors:** Xuanzhen Cen, Datao Xu, Julien S. Baker, Yaodong Gu

**Affiliations:** 1 Faculty of Sports Science, Ningbo University, Ningbo 315211, China; cenxuanzhen@outlook.com (X.C.); andywutong@foxmail.com (D.X.); 2 Department of Sport and Physical Education, Hong Kong Baptist University, Hong Kong, China; jsbaker@hkbu.edu.hk

**Keywords:** arch stiffness index, unplanned gait termination, gait stop, impulse, arch structure

## Abstract

The purpose of this study was to determine relationships between arch stiffness and relative regional impulse during walking, running, and stopping. A total of 61 asymptomatic male subjects volunteered to participate in the study. All were classified by calculating the arch stiffness index using 3-dimensional foot morphological scanning. Plantar pressure distribution data were collected from participants using a Footscan pressure platform during gait tests that included walking, running, and gait termination. The stiff arches group (*n* = 19) and flexible arches group (*n* = 17) were included in the following data analysis. The results suggested that subjects with stiffer arches had a larger and smaller percentage of plantar impulse in the forefoot and rearfoot, respectively, than subjects with more flexible arches during walking and running. However, during gait termination, which included planned and unplanned gait stopping, the plantar impulse distribution pattern was found to be reversed. The current findings demonstrate that the distributional changes of plantar loading follow unidirectional transfer between the forefoot and the rearfoot on the plantar longitudinal axis. Moreover, the patterns of impulse distribution are also different based on different gait task mechanisms.

## 1. Introduction

The human foot arch is the elastic and constrictive cambered structure comprising of tarsal bones, metatarsal bones, and surrounding ligaments and tendons. In general, the anterior transverse arch and the medial and lateral longitudinal arches together form this arch structure. Moreover, the medial longitudinal arch is considered as a significant feature that is different from other primates in the process of evolution [1,2]. The most important contribution of the longitudinal arch in human locomotion, such running and jumping, is to absorb loading impact and to improve the efficiency of ambulation [3,4]. The windlass mechanism of the human foot illustrates well the positive effects of the longitudinal arch in the push-off phase during gait [2,5]. The active elastic subsystem composed of the intrinsic and extrinsic muscles on the foot plays an important role in maintaining arch stability and foot motion [6]. The plantar intrinsic muscles support the foot arches in dynamic activities and have synergistic and modulating effects on the foot arches [6,7]. The extrinsic muscles provide both absorption and propulsion abilities to the longitudinal arch during gait [6]. The difference in arch type is a contributory factor in sports injuries, which could affect and influence physical performance [3,8]. Furthermore, poor exercise behaviors might also lead to progressive deformation in arch morphology, resulting in foot problems and affecting the quality of life [1,9,10,11]. López-López et al. [11] indicated that patients with foot problems present a negative impact on the quality of life related to podiatry and foot health, especially in women.

Foot morphological indexes represented by arch height have also been reported to affect health-related quality of life [10,12]. In the past two decades, arch height index (AHI) has been recognized as a valid and reliable indicator for evaluating arch structure [3,4,8,12], which was outlined by Williams and Mcclay in 2000 [13]. Nevertheless, Wendi and Justin [14] proposed that it is not recommended to use the pure AHI to classify foot arch types due to the interference of ethnicity, gender, and other factors. Moreover, in the course of human movement, the arch of the foot effectively transfers power between the lower limb and the ground through the interaction between the facet joints. In the stance phase, the foot arch will undergo a process of compression–recoil. Meanwhile, the impact load will be absorbed as elastic strain energy and then released at the terminal stance to improve gait efficiency [2,5]. Therefore, the static foot height index alone may not be able to reveal fully the characteristics of dynamic gait.

The arch stiffness index (ASI) can effectively reflect the dynamic load adaptability of the arch by comparing the arch height difference between the standing and sitting posture of the human body and is also used as an effective risk assessment index for physical activities [4]. According to previous studies, there are different methods used to calculate arch stiffness (or arch rigidity, arch flexibility, etc.), all of which are essentially based on changes in subjects’ arch height between the sitting and standing positions [4,15,16,17]. Zifchock et al. [4] found that there were no significant differences in the AHI between male and female subjects, but the arches of female subjects were more flexible than those of male subjects. In addition, AHI of the dominant foot was significantly higher than that of the non-dominant foot, while the ASI showed no significant difference. There was a significant but weak correlation between the AHI and ASI (*p* < 0.01, R^2^ = 0.09). Moreover, in another study [16], subjects with stiffer arches revealed a larger proportion of lateral ground reaction force during running than subjects with a more flexible arch, and the authors proposed that arch flexibility can be used as a criterion to evaluate injury susceptibility.

In addition to walking and running, gait termination is also common in human daily life and physical activity, which reflects the stopping process of the center of mass during moving. A net braking impulse (braking force − push-off force) needs to be provided to adapt a new body posture [18]. During shifting from a dynamic balance to static state (or vice versa), elderly people and patients with balance disorders such as parkinsonism always tend to be at greater risk for injury [19,20]. Especially during unplanned gait termination, the ability to perform termination tasks quickly and effectively in response to unknown stimuli is critical [21]. The consequences of failure are often a fall or even more serious injury. A previous study [22] has reported that unplanned gait termination resulted in larger plantar loading on the lateral metatarsal and heel than planned gait termination, but the role of the arch in this process is still unclear.

Therefore, the aim of this study was to consider how arch stiffness affects the distribution of relative regional impulse (RI_R_) between forefoot, midfoot, and rearfoot during walking, running, and gait termination, respectively. It was hypothesized that the RI_R_ distribution would be different due to the stiffness in the foot arch.

## 2. Materials and Methods

### 2.1. Subjects

A total of 66 male college students volunteered to participate in the study. Of these, 5 (7.58%) were excluded from the experiments after foot morphology scanning revealed abnormal foot morphology, leaving 61 participants (92.42%) for the final analyses. All the participants reported no disorders and injuries to their lower limbs in the first half of the year. Participants were fully familiarized with testing procedures prior to experimental data collection and provided written informed consent. All static foot structure measurements and dynamic gait task measurements were conducted at Research Academy of Grand Health of Ningbo University from October to December 2019. The ethical committee of Ningbo University approved the study (RAGH20181218).

### 2.2. Static Foot Structure Measurements

Each participant’s three-dimensional (3D) foot morphology was collected using the Easy-Foot-Scan (OrthoBaltic, Kaunas, Lithuania), with resolution of 1.0 mm, smoothing of 30 mm, and hole filling of 100 mm [23]. The technology of 3D surface scanning has been validated to have advantages of reliability [23,24]. All subjects were asked to complete a foot scan under static, standing, and sitting conditions, respectively, and keep their legs separated at a shoulder’s width apart. Only the dominant foot (right foot) of all subjects was included in the measurements.

### 2.3. Dynamic Gait Task Measurements

**Figure 1:**
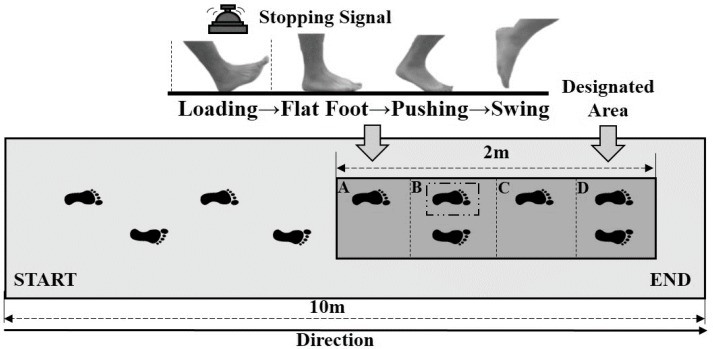
Overhead view of runway used for gait experiments.

After completion of foot morphological scanning, all subjects were required to perform gait tests, including walking, running, and gait termination, conducted on a runway. The runway contained an integrated Footscan plantar pressure plate system (RSscan International, Olen, Belgium) (Figure 1). Each subject was given five minutes to familiarize themselves with the laboratory environment and to warm up.

Firstly, subjects performed barefoot walking and running using their normal comfortable pace to reveal natural gait characteristics on the runway. When more than one whole right foot pattern was measured on the pressure plate the run was considered as a successful trial.

Subjects were then instructed to perform two types of gait termination tests on the runway. The two-meter-long plantar pressure plate was artificially divided into four sections (A, B, C, and D), and every section was about 50 cm × 50 cm. Their primary task was to use the non-dominant leg (left leg) and dominant leg (right leg) to pass section A and B, respectively, and finally terminate on the designated area (section D). If participants received the stopping signal, if the heel of the left foot touched section A, as a result, unplanned gait termination needed to be executed to enable subjects to stop quickly on section B (Figure 1). The staff would send the termination instruction to the subjects by randomly ringing a bell, and the probability of ringing was controlled at about 25%.

For all walking, running, and gait termination sessions, there were two-minute rest intervals between two trials to minimize fatigue affecting the experimental results. Five successful trials for each type of gait task (walking, running, planned, and unplanned gait termination) were required to be collected from each subject.

### 2.4. Data Acquisition

**Figure 2:**
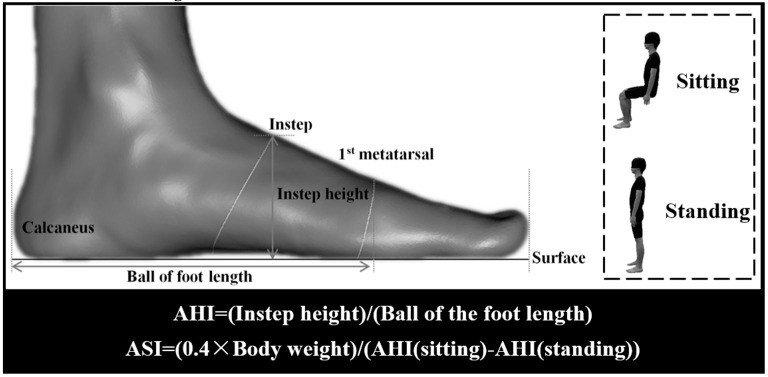
Illustration of foot morphology variables and calculating formulas of AHI (arch height index) and ASI (arch stiffness index).

Based on the two-dimensional foot image obtained from foot morphological scanning, the variables of foot structure were calculated with the AutoCAD software (Autodesk, San Rafael, CA, USA). AHI was measured as the vertical distance from the instep point to surface (instep height) divided by the distance from the first metatarsal joint protrusion to the most posterior point of the calcaneus (ball of the foot length) [12,13]. Each participant’s ASI was calculated by comparing AHI difference between sitting and standing conditions. Zifchock et al. [4,16] indicated that 40% bodyweight (BW) was normalized owing to weight difference supported by the feet in the seated (10% BW) and standing positions (50% BW). Figure 2 outlines detailed foot morphology variables and formulas for calculating AHI and ASI.

Based on the ASI results, all subjects were divided into three groups: stiff arches, normal arches, and flexible arches. The stiff arches and flexible arches groups were defined based on ≥0.5 and ≤0.5 standard deviation from the mean ASI (Table 1). The stiff arches group (*n* = 19) and flexible arches group (*n* = 17) were included in the following data analysis.

The impulse (force–time integral) represents the cumulative effect of plantar force on the stance phase of gait, for characterization of plantar loading on anatomical regions and dynamic foot functions [25]. In this experiment, RI_R_ was selected among the plantar pressure parameters as a representative indicator to assess the plantar loading distribution characteristics during walking, running, and gait termination experiments. This allows local impulse percentage calculations in three parts including rearfoot, midfoot, and forefoot areas accounting for summed impulses, calculated by the following formula:

(1) $${\mathrm{RI}}_{R}\left(\%\right)=\frac{\int^{}F_{i}(t)\mathrm{dt}}{\sum^{}\int^{}F(t)\mathrm{dt}}\times 100.$$

Data on changes in plantar force were also recorded to evaluate the varying pressure patterns. By the Footscan^®^ 7.0 software (RSscan International, Olen, Belgium), the plantar surface was artificially divided into three anatomical regions, including rearfoot, midfoot, and forefoot, and plantar force data were normalized to the initial BW of the subjects.

### 2.5. Statistical Analysis

Prior to experimental data collection, a power of the test analysis was conducted using G*Power version 3.1 software (Franz Faul, Christian-Albrechts-Universität Kiel, Kiel, Germany) and the power value recorded as a result was 0.8. The statistical results were analyzed using SPSS 19.0 for Windows™ software (SPSS Inc., Chicago, IL, USA). Shapiro–Wilks tests were applied to check normal distribution for all variables. Independent-sample t tests were used to analyze the significance of plantar loading distribution between the stiff arches and flexible arches group, and a *p*-value of less than 0.05 was significant for all analyses. In addition, Cohen’s d effect size (ES d) for each variable was also calculated using G*Power. For the effect size, values of 0.2, 0.5, and 0.8 were interpreted as small, moderate, and large, respectively [26].

## 3. Results

**Figure 3:**
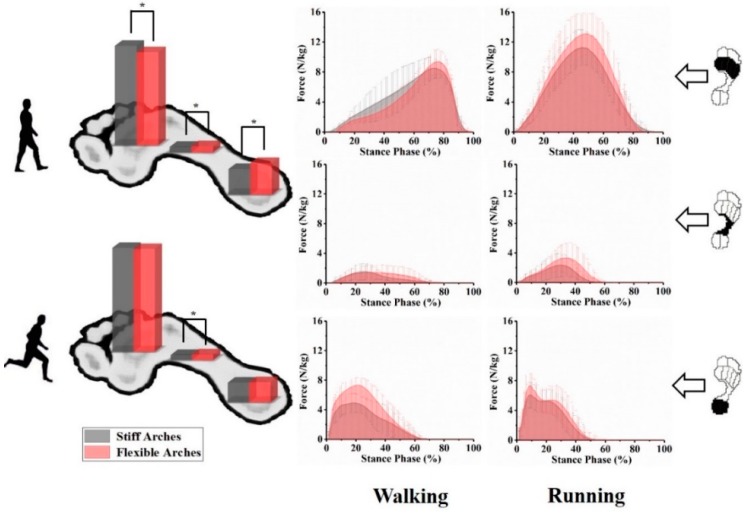
The comparison of impulse distribution between different ASI during walking and running. * Indicates significance with *p* < 0.05.

The plantar pressure data included RI_R_ distributed in the forefoot, midfoot, and rearfoot during walking, running, planned, and unplanned gait termination. Figure 3 shows comparisons of impulse distribution in different ASI groups as well as the change process of plantar force in the stance phase (%) during walking and running. Table 2 presents detailed information of impulse distribution between the stiff arches and flexible arches groups during four dynamic gait tasks.

During walking, compared with individuals with stiff arches, the flexible arches group demonstrated a larger ratio of impulse in the midfoot and rearfoot (*p* = 0.013 and *p* < 0.001), respectively. A significant decrease was observed in the flexible arches group compared with the stiff arches group in the forefoot (*p* < 0.001). A similar plantar impulse distribution trend was found during the running task. However, significant differences only were exhibited in the midfoot part (*p* = 0.022).

During planned gait termination, the plantar impulse distribution pattern was found to be reversed compared with that of walking and running tasks. The RI_R_ of the stiff arches group was larger than that of the flexible arches group in the rearfoot and midfoot (*p* < 0.001, *p* < 0.001). Meanwhile, in the forefoot, the RI_R_ was significantly smaller than that of individuals with flexible arches (*p* < 0.001). In addition, during planned gait termination, significantly larger and smaller RI_R_ was found between rearfoot and forefoot, respectively, when compared with more flexible arches (*p* < 0.001, *p* < 0.001). Although the ratio of impulse of the flexible arch was larger than that of the stiff arch in the midfoot part, the difference was not significant.

## 4. Discussion

During the stance phase of gait, the foot arch will deform owing to supporting the body weight and absorbing any impact. More flexible arches may contribute to the transfer of the resulting impulse to reduce local load in the longitudinal axis. However, our results provide unique insight into the effect of foot arch stiffness on plantar impulse distribution during gait tasks. The distributional change of plantar impulse is observed to follow a unidirectional transfer between the forefoot and the rearfoot on the plantar longitudinal axis. Moreover, the pattern of impulse distribution is also different based on different gait tasks (e.g., walking, running, and gait termination).

### 4.1. The Relationship between ASI and Plantar Impulse during Walking and Running

Arch morphological characteristics are primary aspects of evaluating foot structure and are also associated with foot function and injury [12]. The study of Zifchock et al. [17] verified the common belief that flatfeet tend to be more flexible. In addition, a large proportion of high arches were stiffer. Therefore, in relation to the previous conclusion [27,28] that individuals with cavus foot demonstrated increased force–time integral and other plantar pressure indicators in their medial forefoot during walking, plantar loading patterns may differ between subjects with stiff and flexible arches. This hypothesis was also consistent with results in our study, which showed that subjects in the stiffer arches group experienced a larger proportion of plantar impulse in their forefeet than those in the flexible arches group during walking and running (Figure 3). In addition, in flexible arches, the midfoot and rearfoot parts bear more load than in stiff arches. Similar loading distribution patterns may lead subjects with stiff arches to a propensity toward forefoot problems especially in the metatarsal bone’s sections of the foot [28]. According to loading patterns, more flexible arches may decrease potential injury risk of developing metatarsal stress fractures [29].

It was previously believed that flexible arches were better for splaying in stance phases, shifting the midfoot loading forward and backward concurrently on the longitudinal axis [16]. However, our results are consistent with the findings of Zifchock et al. [16] and contradicted the abovementioned conclusion. In both walking and running tests, stiff arches showed a larger and smaller proportion of total plantar impulse in the forefoot and rearfoot parts, respectively, than flexible arches. The unidirectional impulse transfer on the plantar longitudinal axis could be related to the irregular, asymmetrical triangular truss model formed by the bones of the arch and plantar fascia [30]. The relatively short proximal triangular side firmly attaching the calcaneal tuberosities may bear more impulse during compression of the arch.

### 4.2. The Relationship between ASI and Plantar Impulse during Gait Termination

Interestingly, the study found that the impulse distribution pattern in the two types of gait termination tests was the opposite of that in walking and running (Table 2). Compared with gait movement, the crucial difference in gait termination is that the propulsive phase was weakened [20,31], which may limit the forward splay of the stiff arches. However, the calcaneus is the anatomical structure of the foot that contacts initially with the ground after the terminal swing phase. Most of the body mass is loaded during this phase, which may be the primary cause that the larger proportion of impulse was observed on the rearfoot part of the foot [32]. At the same time, during gait termination, both the stiff and flexible arches groups will experience larger plantar loading than walking and running, which was also verified in our experimental results [22]. The more flexible arches may cushion the impact of the rearfoot during quick and unexpected gait termination stops. Gait stability is often a key aspect in gait termination research, and unplanned gait termination is widely regarded as a great challenge to human stability, especially for elderly people and patients with balance disorders [20,22,33,34]. To the best of our knowledge, there is little arch related research about gait termination at present. Stephen and colleagues [35] found that the midsole material of footwear could affect dynamic balance during unplanned gait termination. They also indicated that footwear with soft midsole material would increase the range of the center of mass position relative to the lateral base of support, resulting in an impairment of the dynamic balance control system.

### 4.3. Clinical Relevance

It was reported that arch stiffness could be applied as a criterion for evaluating injury susceptibility [16]. Current findings also indicated plantar impulse distribution differences and potential injury biases that exist between individuals with different arch stiffnesses. The information may be important to consider in the usefulness of both intrinsic manipulations (such as foot muscle training, foot function prediction, or foot surgical intervention) and extrinsic manipulations (such as the development of arch orthotics or specific types of footwear). Additionally, further studies may involve a more diverse population of subjects or apply various measurements to describe arch stiffness.

### 4.4. Limitations

Several limitations should be noted in relation to this study. Firstly, RI_R_ as the sole plantar pressure variable was selected and used. RI_R_ as a parameter could not control the confounding effects of body weight, foot size, and other individual differences [25]. There was a linear dependence among plantar pressure indexes (peak pressure, mean pressure, and pressure–time integral, etc.) [28,36]. Secondly, the grouping of subjects in this study may need consideration because of the correlation between arch height index and arch stiffness index [17,37]. Finally, the plantar anatomical surface was divided into only three parts, which was originally done to study the distribution of plantar impulse along the longitudinal axis. However, this may cause the medial–lateral distribution between the parts to be ignored. For example, the second and third metatarsal may have less freedom of movement owing to being wedged between the cuneiform joints. This may have contributed to an increase in plantar pressure compared to other metatarsal bones [28]. Further experimental studies could investigate suggestion further.

## 5. Conclusions

The present study indicated the distributional change of plantar impulse is observed to follow a unidirectional transfer between the forefoot and the rearfoot on the plantar longitudinal axis. Moreover, the pattern of impulse distribution is also different based on different gait tasks. These preliminary findings provide further insight into the usefulness of arch stiffness being used as a criterion to assess injury susceptibility.

## Figures and Tables

**Table 1:** The anthropometry characteristics of different groups of subjects’ ASI.

Characteristic	Total	Groups		*p* Value *
		Stiff arches	Flexible arches	
**Number (n)**	61	19	17	NA
**Age (y)**	23.40 ± 0.82	23.66 ± 1.03	23.50 ± 0.55	0.736
**Height (cm)**	179.20 ± 4.67	182.33 ± 4.13	177.33 ± 4.32	0.068
**Weight (kg)**	73.85 ± 7.11	75.83 ± 2.32	72.33 ± 5.54	0.193
**Body mass index (kg/m^2^)**	22.97 ± 1.72	22.82 ± 0.66	23.00 ± 1.56	0.797
**Foot length (mm)**	262.75 ± 4.99	265.00 ± 4.47	262.50 ± 4.18	0.341
**ASI**	1557.53 ± 464.40	2153.99 ± 345.26	1154.67 ± 131.19	0.000*

Note: ASI: arch stiffness index. *: A significant difference between two groups, *p* < 0.05.

**Table 2:** The relative regional impulse (RI_R_) (%) during dynamic gait tasks in stiff and flexible arches groups, including mean, standard deviation (SD), 95% confidence intervals (CI), *p* value, and effect sizes (ES) (Cohen’s d).

RI_R_ (%)		Stiff arches		Flexible arches		95% CI	*p* Value	ES *d*
		Mean	SD	Mean	SD			
**Walking**	rearfoot	19.57	5.12	24.10	3.69	(−5.85,−3.22)	0.000 *	1.02
	midfoot	4.30	1.12	4.80	1.54	(−0.90,−0.11)	0.013 *	0.37
	forefoot	76.03	5.43	71.09	4.13	(3.52,6.36)	0.000 *	1.02
**Running**	rearfoot	15.26	2.91	15.63	2.84	(−1.22,0.48)	0.389	0.13
	midfoot	3.96	1.43	4.46	1.48	(−0.93,−0.07)	0.022 *	0.34
	forefoot	80.78	3.29	79.91	3.67	(−0.16,1.90)	0.096	0.25
**PGT**	rearfoot	29.18	11.46	24.35	5.43	(2.18,7.47)	0.000 *	0.54
	midfoot	8.10	2.23	5.98	1.61	(1.55,2.70)	0.000 *	1.09
	forefoot	62.67	12.24	69.84	6.39	(−10.05,−4.29)	0.000 *	0.73
**UGT**	rearfoot	26.65	7.37	22.12	5.23	(2.65,6.41)	0.000 *	0.71
	midfoot	4.58	1.24	4.67	1.46	(−0.48,0.31)	0.680	0.07
	forefoot	68.77	7.22	73.22	5.47	(−6.33,−2.56)	0.000 *	0.69

Note: RI_R_: relative regional impulse, PGT: planned gait termination, UGT: unplanned gait termination. * A significant different between two groups, *p* < 0.05.
